# Modeling essential connections in obsessive–compulsive disorder patients using functional MRI

**DOI:** 10.1002/brb3.1499

**Published:** 2020-01-01

**Authors:** Xiaodan Xing, Lili Jin, Qingfeng Li, Qiong Yang, Hongying Han, Chuanyong Xu, Zhen Wei, Yiqiang Zhan, Xiang Sean Zhou, Zhong Xue, Xu Chu, Ziwen Peng, Feng Shi

**Affiliations:** ^1^ Medical Imaging Center Shanghai Advanced Research Institute Shanghai China; ^2^ Shanghai United Imaging Intelligence, Co., Ltd. Shanghai China; ^3^ University of Chinese Academy of Sciences Beijing China; ^4^ Center for the Study of Applied Psychology South China Normal University Guangzhou China; ^5^ School of Biomedical Engineering Southern Medical University Guangzhou China; ^6^ Affiliated Brain Hospital of Guangzhou Medical University Guangzhou China; ^7^ Department of Psychiatry The Third Affiliated Hospital Sun Yat‐Sen University Guangzhou China; ^8^ Department of Child Psychiatry Shenzhen Kangning Hospital Shenzhen Mental Health Center Shenzhen China; ^9^ Department of Child Psychiatry The Affiliated Shenzhen Maternity & Child Healthcare Hospital Southern Medical University Shenzhen China

**Keywords:** classification, global brain functional connectivity, obsessive–compulsive disorder

## Abstract

**Object:**

Obsessive–compulsive disorder (OCD) is a mental disease in which people experience uncontrollable and repetitive thoughts or behaviors. Clinical diagnosis of OCD is achieved by using neuropsychological assessment metrics, which are often subjectively affected by psychologists and patients. In this study, we propose a classification model for OCD diagnosis using functional MR images.

**Methods:**

Using functional connectivity (FC) matrices calculated from brain region of interest (ROI) pairs, a novel Riemann Kernel principal component analysis (PCA) model is employed for feature extraction, which preserves the topological information in the FC matrices. Hierarchical features are then fed into an ensemble classifier based on the XGBoost algorithm. Finally, decisive features extracted during classification are used to investigate the brain FC variations between patients with OCD and healthy controls.

**Results:**

The proposed algorithm yielded a classification accuracy of 91.8%. Additionally, the well‐known cortico–striatal–thalamic–cortical (CSTC) circuit and cerebellum were found as highly related regions with OCD. To further analyze the cerebellar‐related function in OCD, we demarcated cerebellum into three subregions according to their anatomical and functional property. Using these three functional cerebellum regions as seeds for brain connectivity computation, statistical results showed that patients with OCD have decreased posterior cerebellar connections.

**Conclusions:**

This study provides a new and efficient method to characterize patients with OCD using resting‐state functional MRI. We also provide a new perspective to analyze disease‐related features. Despite of CSTC circuit, our model‐driven feature analysis reported cerebellum as an OCD‐related region. This paper may provide novel insight to the understanding of genetic etiology of OCD.

## Graphical abstract

This paper proposed a method based on kernel PCA and Riemann manifold to characterize patients with OCD from healthy controls, whose performance reaches 90%. Furthermore, by feature reconstruction, our algorithm could generate decisive features from the classification model. Results demonstrate the possible affect of cerebellum in the etiology of OCD.

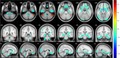

## 1 INTRODUCTION

Obsessive–compulsive disorder (OCD) is a mental disorder with an approximate lifetime prevalence of 1%–3% (Angst et al., 2005). Patients suffering from obsessions have persistent intrusive thoughts, and patients suffering from compulsions have repetitive behaviors. Despite of these symptoms and their impairment to patients' social functioning, it is highly possible that OCD can rise to a wide spectrum of additional psychiatric disorders, including major depressive disorder (MDD), tics, and panic disorder (PD; Angst et al., 2005; Ruscio, Stein, Chiu, & Kessler, 2010).

Clinically, the diagnosis of OCD is achieved by (a) neuropsychological metrics, such as YBOCS (Goodman, Price, Rasmussen, Mazure, Delgado, et al., 1989; Goodman, Price, Rasmussen, Mazure, Fleischmann, et al., 1989), Obsessive–Compulsive Inventory (OCI; Foa et al., 2002), and CY‐BOCS for children (Scahill et al., 1997), (b) physical tests, such as complete blood count and alcohol/drug tests, and (c) interview by psychologists. However, such diagnosis methods are easily affected by psychologists' subjection, and symptoms of comorbidities also interfere with the diagnosis. Thus, a more objective diagnosis model is desirable for accurate and robust measurements.

Decisive features, which could be used for OCD classification, can be extracted from both structural MRI and functional MRI. Structural MRI often detects morphological abnormality or provides radiomic features. Although many works have reported significant volumetric differences between patients and healthy controls (Gilbert et al., 2008; Hu et al., 2017; Peng et al., 2012), they focus on local volumetric variability and neglect the integrity of the brain. Functional MRI measures brain activity based on blood‐oxygen‐level‐dependent level (BOLD) signals. Previous works have shown that onset of OCD demonstrates functional abnormalities across different anatomical regions. In this study, we focus on functional MRI and study connectivity‐based classification for OCD diagnosis.

In the literature, neurological dysfunction in OCD brains has been studied extensively. Harrison et al. (2009) used resting‐state functional MRI and found abnormal activation in connections between striatum and orbitofrontal cortex. A meta‐analysis by Guersel, Avram, Sorg, Brandl, and Koch (2018) revealed disrupted fronto‐stratal circuits and impaired large‐scale fronto‐parietal‐limbic brain networks in patients with OCD. Further, cortico–striatal–thalamic–cortical (CSTC) circuit has been identified as a decisive imaging marker for OCD diagosis. For example, Beucke et al. (2013) showed that distant connectivity of the orbitofrontal cortex and the putamen positively correlates with the severity of OCD symptoms. Sakai et al. (2011) investigated the corticostriatal functional connectivity in nonmedicated OCD patients and reported an increased connectivity associated with the ventral striatum in the orbitofrontal cortex, ventral medial prefrontal cortex, and dorsal lateral prefrontal cortex in OCD.

Functional connectivity (FC) matrix, also known as connectome, was computed by the correlations between each brain region with all other regions. Conventional connectome classification algorithms treat a connectome as a vector of features and then feed it into “off‐the‐shelf” classifiers like support vector machine (SVM) and decision tree (Castellanos, Martino, Craddock, Mehta, & Milham, 2013). However, such methods discard the topological structure of connectivity matrix and may lose useful anatomical information in the connectomes. To remedy this drawback, considering the graphical nature of connectivity, graph kernel‐based classifiers have been applied on inter‐subject discrimination of two different types of auditory stimuli (Vega‐Pons, Avesani, Andric, & Hasson, 2014) and classification of schizophrenia (SZ) and healthy control (HC; Zhou, Mei, Li, & Huang, 2017). Other graphical theory‐based methods investigating multi‐spectrum networks (Wee et al., 2012), ordinal relationship of edges of FC matrices (Zhang et al., 2018), brain network embedding algorithms (Cao et al., 2017), and graph convolution neural networks (Ktena et al., 2017) are also proposed.

Using linear statistical analysis methods, such as Student's *t* tests, to compare the magnitude of functional connectivity between groups, significant group difference between patients with OCD and healthy controls could be discovered. CSTC circuit has been implicated in OCD pathophysiology by functional connectivity findings (Beucke et al., 2013; Markarian et al., 2010; Sakai et al., 2011). However, such group comparison methods ignore the underlying hierarchical and spatial information of functional connectivity matrices. Moreover, significantly different connections by themselves may not produce feasible diagnostic yields.

To address aforementioned problems, we propose a novel connectome decomposition algorithm called Riemann manifold principal component analysis (PCA) for efficient feature extraction from functional connectivity matrices. We hypothesize that artificial intelligence algorithms can help diagnose OCD and can extract significant abnormalities in fMRI. We tested our classification algorithms on 128 subjects, 61 of which are patients with OCD, and we also reported the most useful connections in this classifier. As a result, in addition to the CSTC circuit, cerebellum was also reported as an important region during the classification. The cognitive function of cerebellum was rarely investigated in previous studies. To analyze the functional effect of cerebellum on OCD, we performed a post hoc seed‐based analysis and identified posterior cerebellum as an affected region.

In summary, the contribution of this paper is threefold: (a) a feature extraction algorithm based on the manifold property of functional connectivity matrices; (b) a model‐driven approach to detect discriminant imaging markers for OCD diagnosis; and (c) a seed‐based analysis on three functional subregions of cerebellum, investigating the cerebellar functioning in patients with OCD.

## 2 METHODS

**Figure 1:**
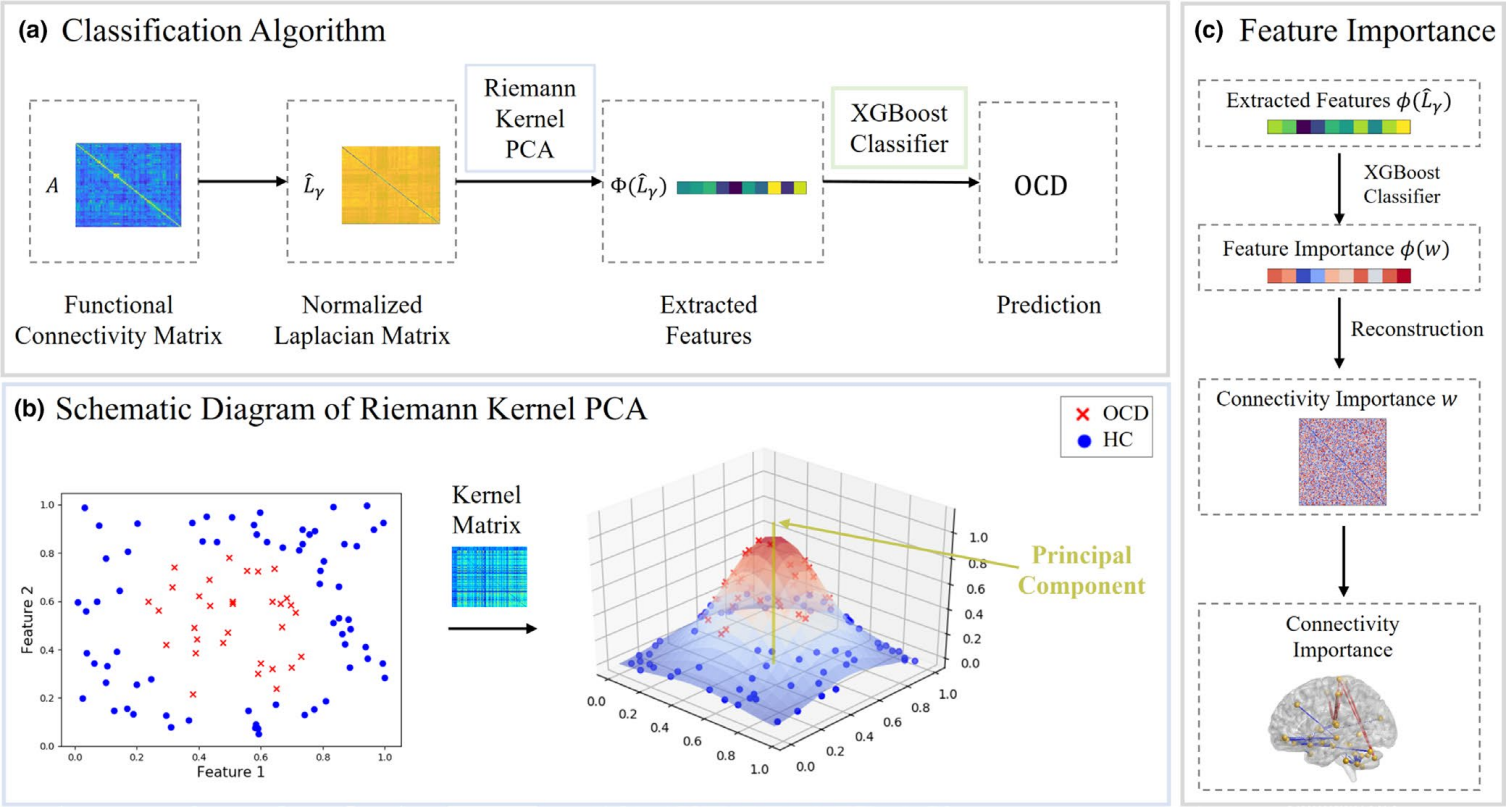
Flowcharts of the proposed disease diagnosis algorithm by using Riemann Kernel PCA for feature extraction. Part (a) is the main flowchart. Connectivity matrices were first normalized as their graph Laplacians. Using proposed Riemann Kernel, we could generate the kernel matrix which describing the geodesic distances among all subjects. By selecting the eigenvalues of kernel matrix, PCA was performed on FC matrices. The extracted features were sent to XGBoost for disease diagnosis. Part (b) presents a schematic representation of Riemann kernel PCA. Using kernel trick, nonlinearly distributed data points could be projected onto its principal components. Part (c) illustrates that we could retrieve decisive features from XGBoost classifier by using pre‐image based kernel PCA reconstruction algorithm

The schematic representation of our classification method is shown in Figure 1. Functional connectivity matrices are composed of functional correlations between each ROI pair of the brain. First, connectivity matrices are normalized into their graph Laplacians. Then, kernel matrices are computed by using the Riemannian log‐Euclidean distance between each subject. By analyzing the eigenvectors corresponding to a number of largest eigenvalues, we could derive the principal components of our data. By projecting original high dimensional features into these principal components, decomposed features could be obtained. Third, decomposed features are fed into a classification model based on a decision tree. Finally, in order to find decisive imaging markers for OCD diagnosis, we implement a feature reconstruction algorithm to retrieve important features from the classifier.

### 2.1 Data acquisition and preprocessing

Neuroimaging data were obtained from Huiai Hospital of Shenzhen. The dataset includes 67 healthy controls and 61 patients, with age ranging from 18 to 59 years. Written consents were obtained from all participants. The demographic information and clinical characteristics are shown in Table 1. Univariate ANOVA (analysis of variance) was carried out to make sure these two groups have no significant age difference. All patients enrolled passed the test defined by the Diagnostic and Statistical Manual of Mental Disorders, 4th edition (DSM‐IV) criteria for OCD by using the Structured Clinical Interview (SCID) for DSM‐IV‐TR Axis I disorders (First, Spitzer, Gibbon, & Williams, 2002). The exclusion criteria are as follows: (a) if they were younger than 18 or older than 59 years; (b) if they had a history of brain trauma or neurological disease; and (c) if they had shown alcohol/substance abuse within 12 months prior to participation. Healthy controls were screened by using the SCID for the DSM‐IV‐TR Axis I disorders, Research Version, Non‐Patient edition (SCID‐I/NP; Spitzer, Robert, Gibbon, & Williams, 2002). Healthy controls with a family history of axis I or II psychiatric disorders were excluded.

**Table 1:** Demographic and clinical characteristics information of patients (OCD) and healthy controls (HC)

	OCD	HC
Demographic measures		
Number of male (Female) subjects	61 (45)	67 (44)
Age (Mean ± *SD*)	26.1 ± *8.1*	21.3 ± *5.0*
Clinical measures		
YBOCS total (*SD*)	25.5 ± *7.0*	2.4 ± *2.9*
YBOCS obsessions (*SD*)	15.4 ± *3.1*	0.9 ± *1.3*
YBCOS compulsions(*SD*)	10.9 ± *4.8*	1.4 ± *2.1*

Note

The average and standard deviation of continuous variable are provided, of which standard deviation is shown italic. Compulsion and obsession were measured, respectively, by YBOCS (Yale–Brown Obsessive‐Compulsive Scale). According to *t* tests, there are no significant age difference (*p* = .06) among three groups.

Structural images were acquired with TR = 8 ms, TE = 1.7 ms, flip angle = 20°, and resolution = 1.0 × 1.0 × 1.0 mm^3^. Functional MR images were obtained with TR = 2,000 ms, TE = 60 ms, flip angle = 90°, resolution = 3.75 × 3.75 × 4.0 mm^3^, and 33 sagittal slices with 230 time points. T1 images were bias‐corrected and skull‐stripped. Each subject's structural scan was registered to the standard template in the Montreal Neurological Institute (MNI) space and parcellated into 116 regions by Automated Anatomical Labeling (AAL; Tzourio‐Mazoyer et al., 2002) template. Functional images were preprocessed by DPARSF 4.3 (Data Processing Assistant for Resting‐State fMRI, advanced edition; Yan & Zang, 2010), and preprocessing steps include slice time correction, motion correction, intensity normalization, spatial and temporal filtering, and nuisance covariates regression. In particular, first 10 time points were removed before slice timing. Images were time corrected by interpolation and motion corrected by a rigid body transformation on each volume. Considering the influence of head motion on fMRI signals (Power, Barnes, Snyder, Schlaggar, & Petersen, 2012; Satterthwaite et al., 2013), images with excessive movement (more than 1 mm translation on *x*‐axis, *y*‐axis, and *z*‐axis and 2° of rotation) and its neighbor volumes were scrubbed (Power et al., 2012). Functional scans were first rigidly transformed to their structural scans and then normalized to the MNI space using the warping parameters from the abovementioned warping procedure. After normalization, Gaussian kernel with 4 mm FWHM (Full Wave at Half Maximum) was used for image smoothing. To reduce noise from hardware and subject's motion, all BOLD time series were filtered by a band‐pass frequency filtering ranging from 0.01 to 0.1 Hz. Three covariates were regressed out: head motion parameters, white matter signal, and cerebrospinal fluid signal.

### 2.2 Feature extraction

Principal component analysis is a popular feature decomposition and extraction algorithm. It maps a *n*‐dimension feature into a *k*‐dimension ***linear*** space (*k* ≤ *n*) formed by principal components (Wold, Esbensen, & Geladi, 1987). Considering a centralized feature matrix *X* = {***x*_1_**, ***x*_2_**, …, ***x_m_***} ϵ *R^n^* ^×^ *^m^* to be decomposed in to $X^{\prime }=\left\{{x^{\prime }}_{1},{x^{\prime }}_{2},\ldots ,{x^{\prime }}_{m}\right\}\in R^{n^{\prime }\times m}$, where *m* denotes the number of subjects and *n* denotes the number of original features, PCA calculates the $n^{\prime }$ largest eigenvalues $\left\{\lambda_{1},\ldots \lambda_{n^{\prime }}\right\}$ and corresponding eigenvectors $U=\left\{u_{1},\ldots u_{n^{\prime }}\right\}\in R^{n\times n^{\prime }}$ of the covariance matrix $C=\frac{1}{n}XX^{T}$ and translates *X* into $X^{\prime }$ by $X^{\prime }=U^{T}X$. The term “centralized” here means the column‐wise mean of *X* is zero.

In the cases when data are confined to a ***nonlinear*** space, kernel trick can be used to model such a nonlinear manifold. Kernel trick defines a higher dimensional space via the inner product of the higher dimensional representations, normally using a Gaussian kernel. By lifting the original data *X* = {***x*_1_**, ***x*_2_**, …, ***x_n_***} into a hyperspace Φ (*X*) = {*ϕ* (***x*_1_**), *ϕ* (***x*_2_**), …, *ϕ* (***x*_n_**)}, the probability of data being linear separatable rises. Thus, we could perform a linear PCA on Φ (*X*) = {*ϕ* (***x*_1_**), *ϕ* (***x*_2_**), …, *ϕ* (***x*_n_**)} and decompose Φ (*X*). Kernel matrix is defined by $K=\Phi {\left(X\right)}^{T}\Phi \left(X\right)$, and the covariance matrix is *C_ϕ_* = Φ (*X*) Φ (*X*)*^T^*. To compute the principal components of *C_ϕ_*,

(1) $$\Phi \left(X\right)\Phi {\left(X\right)}^{T}u=\lambda u$$

by multiplying Φ (*X*)*^T^* on both sides, we get,

(2) Kα=λα

where ***α*** = Φ (*X*)*^T^* ***u*** and $u=\frac{1}{\lambda }\Phi \left(X\right)\alpha$. The normalized eigenvectors are denoted as $\tilde{\alpha }=\frac{1}{||X^{T}u||}X^{T}u=\frac{1}{\sqrt{\lambda }}\alpha$. Thus, by performing eigen‐decomposition on kernel matrix K, we obtain the principal components $\left\{u_{1},u_{2},\ldots ,u_{n^{\prime }}\right\}$ of vectors in the hyperspace.

A widely used kernel function is Gaussian RBF kernel $K=\left\{k_{\mathrm{ij}}|k_{\mathrm{ij}}=e^{-\frac{||x_{i}-x_{j}|{|}^{2}}{2\sigma^{2}}}\right\}$. It uses the Euclidean distance $||x_{i}-x_{j}|{|}^{2}$ to measure the distance of data points between each other. However, the set of matrices does not lie on a Euclidean space. Considering that the graph Laplacian of connectivity matrices is symmetric (semi‐)positive definite (SPD) and the set of SPD matrices forms a Riemannian manifold (Pennec, Fillard, & Ayache, 2006), the distance function could thus be replaced by geodesic distance on Riemann manifold to capture and preserve the topological property. Here, we used log‐Euclidean geodesic distance as the distance between connectivity matrices to apply a Riemann kernel PCA.

Although not being invariant to affine transformations, Log‐Euclidean method has a simple formulation. For matrix *S*_1_ and *S*_2_ in SPD matrix set *S* ^++^, the log‐Euclidean distance of them is:

(3) $$d_{\log E}\left(S_{1},S_{2}\right)={\left|\log\left(S_{1}\right)-\log\left(S_{2}\right)\right|}_{F}$$

where ${\left|\cdot \right|}_{F}$ denotes the Frobenius norm of a matrix. With the aim of working on Riemann manifold, we computed every connectivity matrix's graph Laplacian. Let *A_s_* ϵ *R^k^* ^×^ *^k^* denote the connectivity matrix of subject *s*‐th where *k* is the number of brain ROIs, the graph Laplacian of this connectivity matrix is:

(4) $$L_{s}=D_{s}-A_{s}$$

where *a_i,j_* > 0, *D_s_* = diag (∑*_j_ a_i,j_*) is the degree matrix of *A_s_*. *L_s_* is semipositive definite and can be mapped into a SPD matrix by ${\hat{L}}_{\gamma }=L+\gamma I$, where *γ* > 0. Thus, the Riemann kernel function of matrices can be generated as

(5) $$K\left({\hat{L}}_{\gamma ,1},\;{\hat{L}}_{\gamma ,2}\right)=e^{\left(-\frac{d_{\log E}^{2}\left({\hat{L}}_{\gamma ,1},\;{\hat{L}}_{\gamma ,2}\right)}{2\sigma^{2}}\right)}$$

with this kernel function, we implemented kernel PCA on our raw data to extract hierarchical features.

### 2.3 Discriminant feature retrieving

In this paper, we used the XGBoost classifier to identify the status of patients. XGBoost classifier is based on the boosting tree algorithm. After the boosted tree is constructed, we can retrieve the feature importance scores for each input feature. First, the importance score is calculated on a single decision tree by the improvement of the split on each feature. Gini index (or Gini impurity, measures the probability of mislabeling objects in the dataset) is used to select the split points. Then, the importance score of every features is averaged across all decision trees within the model (Hastie, Tibshirani, Friedman, & Franklin, 2005). For an input feature vector *f* ϵ *R^N^*, boosted tree could provide an importance vector ***w*** ϵ *R^N^*, each element of which is the importance of corresponding feature.

However, because of kernel trick used for feature extraction, the features' input into classifiers is on a hyperspace where the projection Φ from original matrices to corresponding vectors on the space is unknown. To obtain the feature importance on original feature space, which is the connections between brain regions, we applied a kernel PCA reconstruction algorithm based on pre‐images (Schölkopf, Mika, Smola, Rätsch, & Müller, 1998).

Given a matrix of features' importance ***w*** ϵ *R^k^* ^×^ *^k^* for features in original space, whose entries represent the importance of corresponding connection, we define *P* the projection of it onto principal components and rewrite it according to Equation 2:

(6) $$\begin{array}{c} P_{n^{\prime }}\Phi \left(w\right)=\sum_{k=1}^{n^{\prime }}\beta_{k}{\tilde{\alpha }}_{k}\\ \beta ={\tilde{\alpha }}^{T}\Phi \left(w\right)={\left(\frac{1}{\sqrt{\lambda }}X^{T}u\right)}^{T}\Phi \left(w\right)=\frac{1}{\sqrt{\lambda }}u^{T}\left(\begin{array}{c} K\left(w,\;S_{1}\right)\\ \vdots \\ K\left(w,\;S_{n}\right) \end{array}\right) \end{array}$$

where *β* is the feature importance vector in hierarchical feature space and *n*′ is the dimension after decomposition. Kernel PCA has a property that if *n*′ is the rank of K, $P_{n^{\prime }}\Phi \left(w\right)=\Phi \left(w\right)$; and for smaller *n*′, the overall squared error $\sum_{i=1}^{n}{|P_{n^{\prime }}\Phi \left(S_{i}\right)-\Phi \left(S_{i}\right)|}^{2}$ is minimal (Schölkopf, Smola, & Müller, 1997). To approximate ***w***, we are looking for a $\hat{w}$ such that:

(7) $$l\left(\hat{w}\right)={\left|P_{n^{\prime }}\Phi \left(w\right)-\Phi \left(\hat{w}\right)\right|}^{2}$$

is minimized. Expanding (7) and considering that for Riemann kernel PCA, $K\left(w,w\right)=\text{const}.$, the objective function takes the form:

(8) $$l\left(\hat{w}\right)=-\frac{2}{\lambda }\sum_{k=1}^{n^{\prime }}\beta_{k}\sum_{k=1}^{n}\alpha_{i}^{k}K\left(\hat{w},\;S_{i}\right)+C$$

To minimize (8), we find $\hat{w}$ such that $\nabla l\left(\hat{w}\right)=0$. According to kernel function (5), we can learn $\hat{w}$ iteratively using equation below:

(9) $${\hat{L}}_{\omega ,\gamma }^{\text{new}}=\frac{\sum_{i=1}^{n}\delta_{i}^{k}\exp\left(-\frac{d_{\log E}^{2}\left({\hat{L}}_{\omega ,\gamma }^{\text{old}},\;{\hat{L}}_{S_{1},\gamma }\right)}{2\sigma^{2}}\right)S_{i}}{\sum_{i=1}^{n}\delta_{i}^{k}\exp\left(-\frac{d_{\log E}^{2}\left({\hat{L}}_{\omega }^{\text{old}},\;{\hat{L}}_{S_{1}}\right)}{2\sigma^{2}}\right)}$$

${\hat{L}}_{\omega ,\gamma }$ is the Laplacian matrix of $\hat{w}$. $\delta_{i}^{k}=\sum_{k=1}^{n^{\prime }}\beta_{k}\alpha_{i}^{k}$. Since Laplacian matrix has the same nondiagonal entries with original matrix, ${\hat{L}}_{\omega ,\gamma }$ can represent the importance of features in original feature space.

### 2.4 Seed‐based analysis on cerebellum

Despite of the well‐known CSTC circuits, we also found that cerebellum is highly related to OCD. Although in most studies cerebellum was known to be a region controlling body balance and motion, not taking part in any cognitive decisions, the findings of this paper suggest abnormality in functional connection related to the cerebellum. This is also supported by other recent studies. In 2001, Buckner (Buckner, Krienen, Castellanos, Diaz, & Yeo, 2011) analyzed the functional images from more than 1,000 subjects, aiming to find the connections between cerebellum and cerebrum and found that the cerebellum possesses at least two large, homotopic maps of the full cerebrum. Hou et al. (2012) measured neural activity and found declined ALFF (amplitude of low frequency fluctuation) in bilateral cerebellum of patients with OCD.

**Figure 2:**
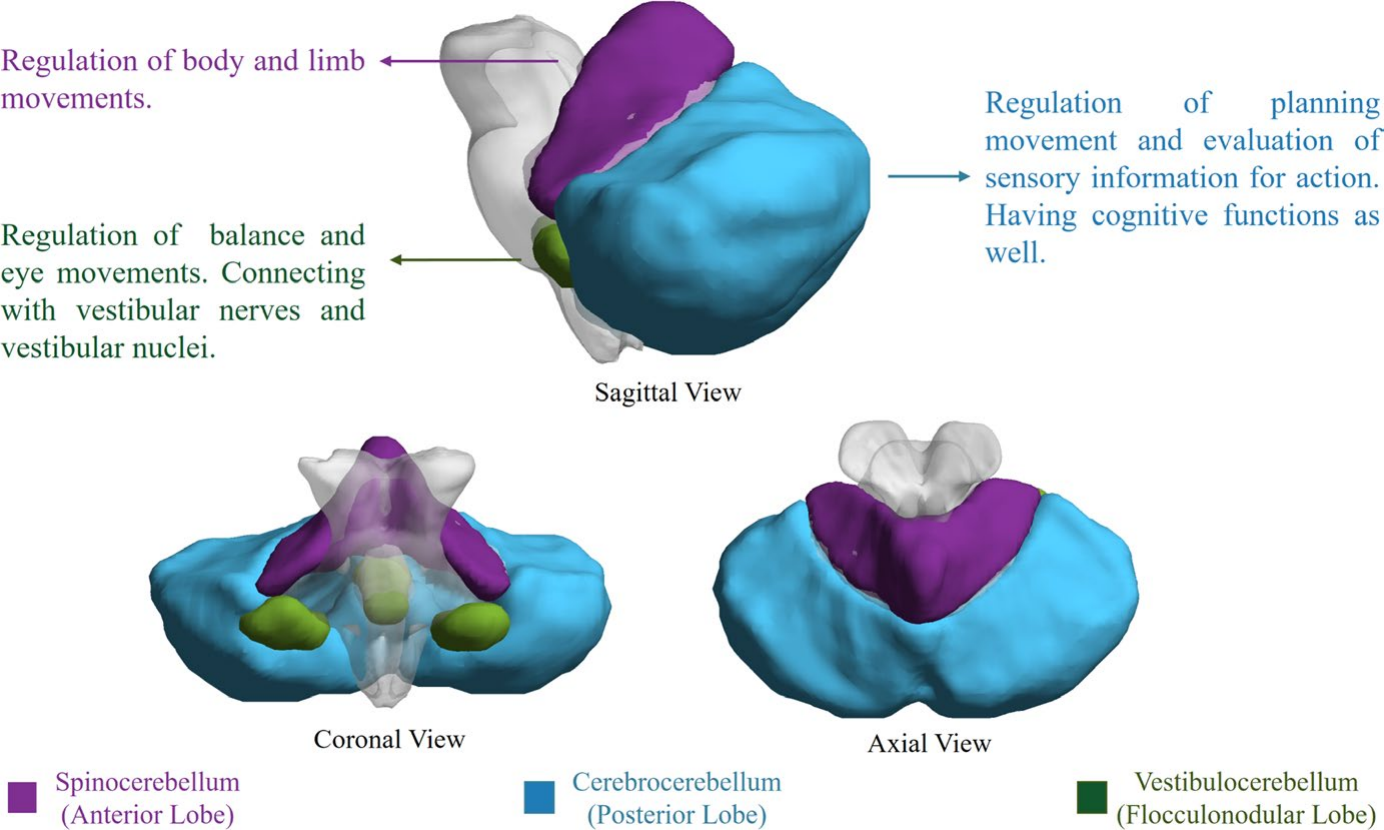
Cerebellar functional subregions and their functions. Vestibulocerebellum mainly receives fiber from vestibular nerves and nuclei, regulating body balance and eye movement. Spinocerebellum controls the movements of trunk muscles. Cerebrocerebellum is considered to have cognitive functions, regulating and coordinating skilled voluntary movement

To fully assess the cerebellum‐related functional networks of OCD, we applied seed‐based correlation analyses on our resting‐state functional MRI dataset. Different from previous studies which considered cerebellum as a whole, we demarcated cerebellum into three anatomical sections, that is, anterior lobule, posterior lobule, and flocculonodular lobule (O'Reilly, Beckmann, Tomassini, Ramnani, & Johansen‐Berg, 2009; Voogd & Glickstein, 1998). The anatomical location of these three subregions is shown in Figure 2. Flocculonodular lobe, also denominated as vestibulocerebellum, is functionally connected with semicircular canals and vestibular nuclei, regulating balance and eye movements. Flocculonodular lobe and other sections are separated by posterolateral fissure. Anterior lobe of cerebellum is also known as spinocerebellum, constituted by central lobe, culmen, uvula, and pyramid of vermis. Spinocerebellum receives input and sends signal back to spinal cord, thus controls body and limb movements. Lateral parts of the cerebellum are cerebrocerebellum, locating between posterolateral fissure and primary fissure. Posterior lobe of cerebellum receives input signal from the cerebral cortex via the pontine nuclei, controlling voluntary movement.

Seed‐based correlation analysis in functional MRI is another widely used method to assess functional connectivity in the brain. Correlation is calculated between the averaged time series of the voxels from the ROI (or the “seed”) and time series from all other voxels. The result of seed‐based analysis is a 3D‐connectivity map indicating the correlation intensity of all other voxels with the seed. Pearson correlations computed by DPARSF are then normalized using a fisher‐Z transform. We applied a *t* test that co‐varies with age and gender, on each subject's correlation map, aiming to find connections varying significantly between two groups.

## 3 EXPERIMENTS

### 3.1 Classification result

**Figure 3:**
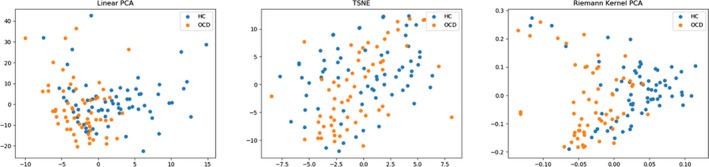
Data structure visualization by linear PCA, Riemann kernel PCA, and t‐SNE (t‐distributed Stochastic Neighbor Embedding). As is shown, our decomposition algorithm could generate a better representation for FC matrices

We evaluated the classification performance by sensitivity, specificity, and accuracy. Sensitivity measures the proportion of real patients that are correctly identified as patients. Specificity measures the proportion of healthy controls that are correctly identified as healthy controls. Accuracy is the proportion of correctly predicted subjects. Decomposition methods using linear PCA and traditional Euclidean kernel PCA are also performed for comparison, as shown in Table 2. Besides, we also visualized the data distribution by reducing the dimension of the FC matrices into two. Results are shown in Figure 3.

**Table 2:** Classification results and comparison

	Sensitivity (%)	Specificity (%)	Accuracy (%)
Pearson's correlation			
SVR	63.9	**95.5**	80.6
XGBoost	86.8	89.7	88.4
Linear PCA			
SVR	72.0	80.3	76.0
XGBoost	75.4	80.1	78.3
Gaussian kernel PCA			
SVR	73.7	75.0	74.4
XGBoost	77.0	73.5	75.2
Riemann kernel PCA			
SVR	86.6	85.1	86.6
XGBoost	**92.6**	90.7	**91.8**

Note

These results are 10‐fold validated. Linear PCA and Riemann kernel PCA models were fitted with training data and then applied on validation data during 10‐fold validation. Bold values are the highest values in each column.

### 3.2 Parameter selection

The Riemann kernel function of matrices can be generated as

$$K\left({\hat{L}}_{\gamma ,1},{\hat{L}}_{\gamma ,2}\right)=e^{\left(-\frac{d_{\log E}^{2}\left({\hat{L}}_{\gamma ,1},\;{\hat{L}}_{\gamma ,2}\right)}{2\sigma^{2}}\right)}$$

**Figure 4:**
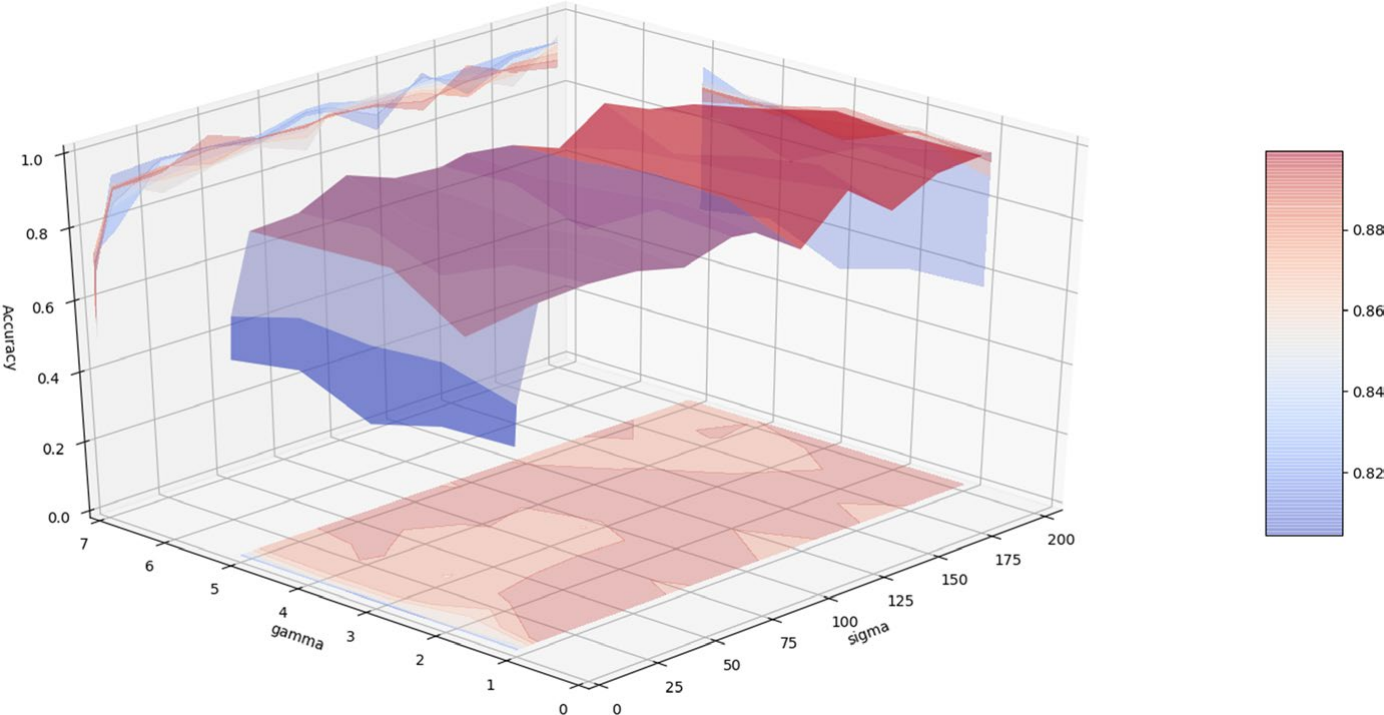
The effect of parameter changing on classification performance. By changing two parameters in Equation 5, different features were extracted and sent into XGBoost classifiers. By measuring the classification performance, we could evaluate the feature extraction performance under different parameter settings. As is shown, the feature extraction performance is not sensitive to regularization parameter *γ*. The distribution parameter *σ* reflects the variance in the set of functional matrices, and smaller or larger choice could compromise the feature extraction performance

Two parameters affect the performance of kernel PCA. *σ* is the scale parameter of kernel function, which represents the standard deviation of the entries of all matrices. Larger *σ* reflects a wider distribution of functional matrices. *γ* is a regularization parameter which regularizes Laplacians to become positive definite because the set of symmetric positive definite matrices is a Riemannian manifold. We compared the performance of Riemann kernel PCA under different parameters. As is shown in Figure 4, the feature extraction performance of Riemann kernel PCA is not sensitive to *γ*, while the best feature extraction performance is reached when *σ* is near the standard deviation of the entries from all matrices.

### 3.3 Decisive features

**Figure 5:**
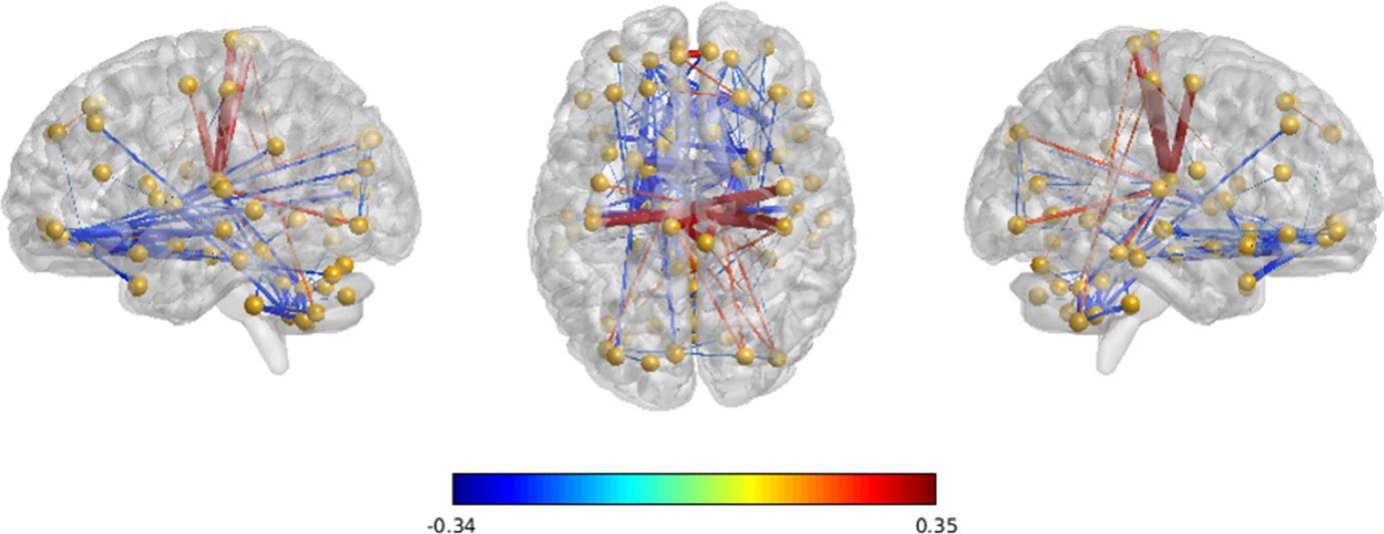
Group difference of functional connectivity for discriminant connections observed between OCD patients and Healthy Controls. This image is shown with a threshold of group connectivity absolute difference >0.2. Blue indicates a group‐wise weaker connection of patients with OCD over healthy controls, and red indicates a stronger connection of patients with OCD over healthy controls. The thickness of every connection indicates the relative magnitude of this connection among all connections shown

Important features were found in inner cerebellar connections, especially posterior parts, and cerebellar connections with basal ganglia, shown in Figure 5. Dysfunctions in the connection between right rectus and left parahippocampal gyrus and in the connection between left rectus and right parahippocampal gyrus are also reported. Abnormal increases in connections are found mostly in thalamus–cortex connections, like postcentral cortex, indicating an activity disruption in thalamus, which possible may contribute to OCD's comorbidity with anxiety and depression (Greicius et al., 2007; Sturm et al., 2003).

### 3.4 Seed‐based analysis on cerebellum

**Figure 6:**
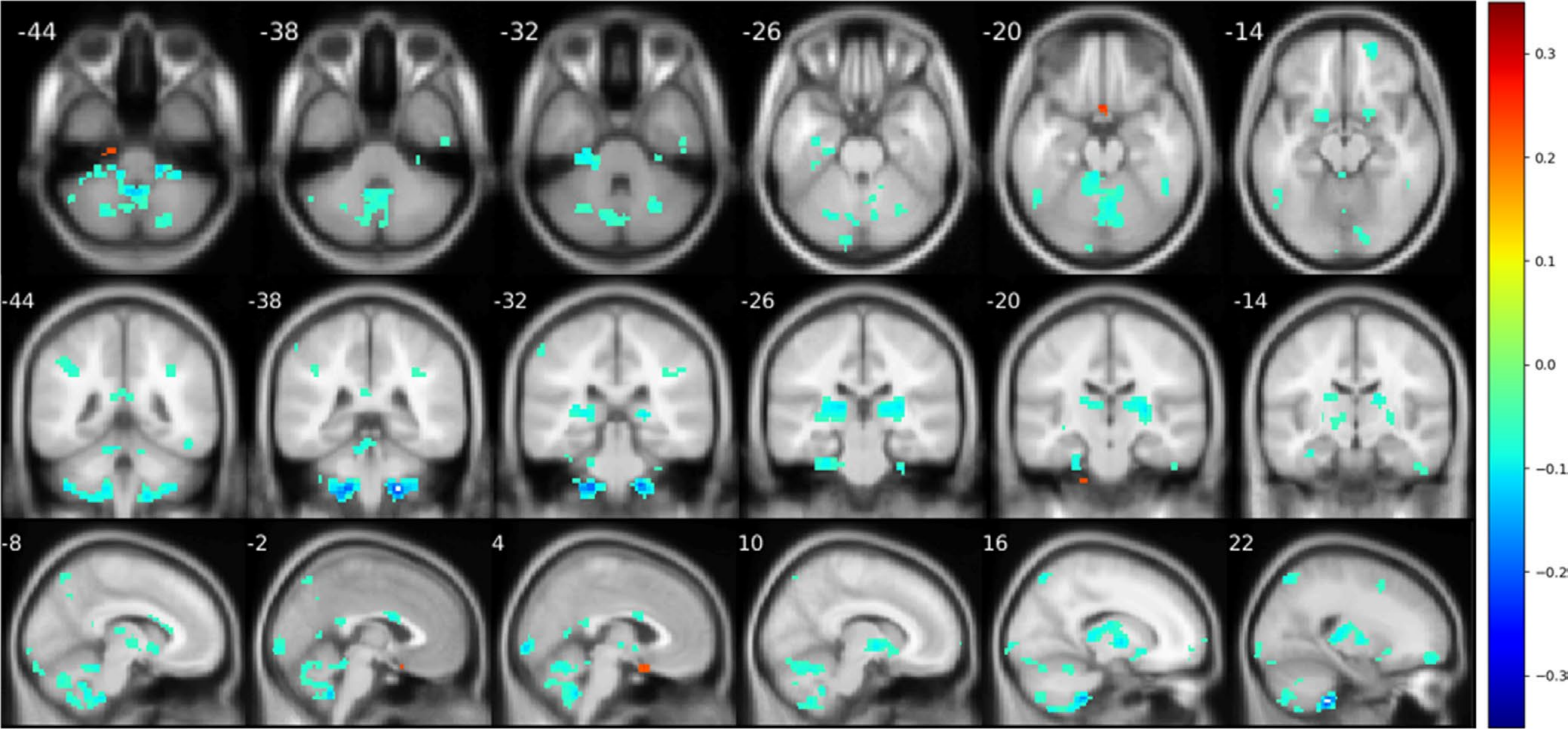
Significant difference (*p* < .05) reported from seed‐based analysis with seed in flocculonodular lobe. Images are shown in a threshold of an absolute value 0.1 for the mean difference between two groups. For vestibulocerebellum, abnormal decline in connections was found in thalamus and other cerebellum regions

**Figure 7:**
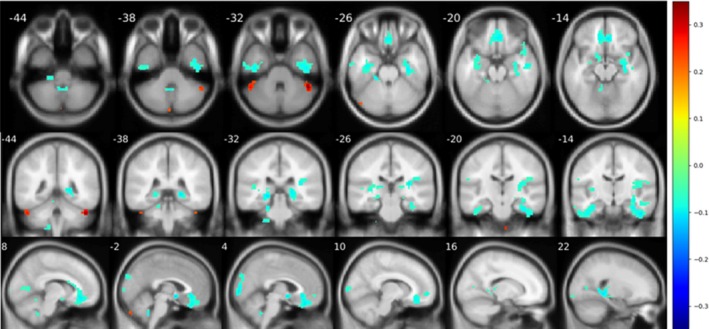
Significant difference (*p* < .05) reported from seed‐based analysis with seed in anterior lobe. Images are shown in a threshold of an absolute value 0.1 for the mean difference between two groups. A noticeable decline is found in rectus and right insula

**Figure 8:**
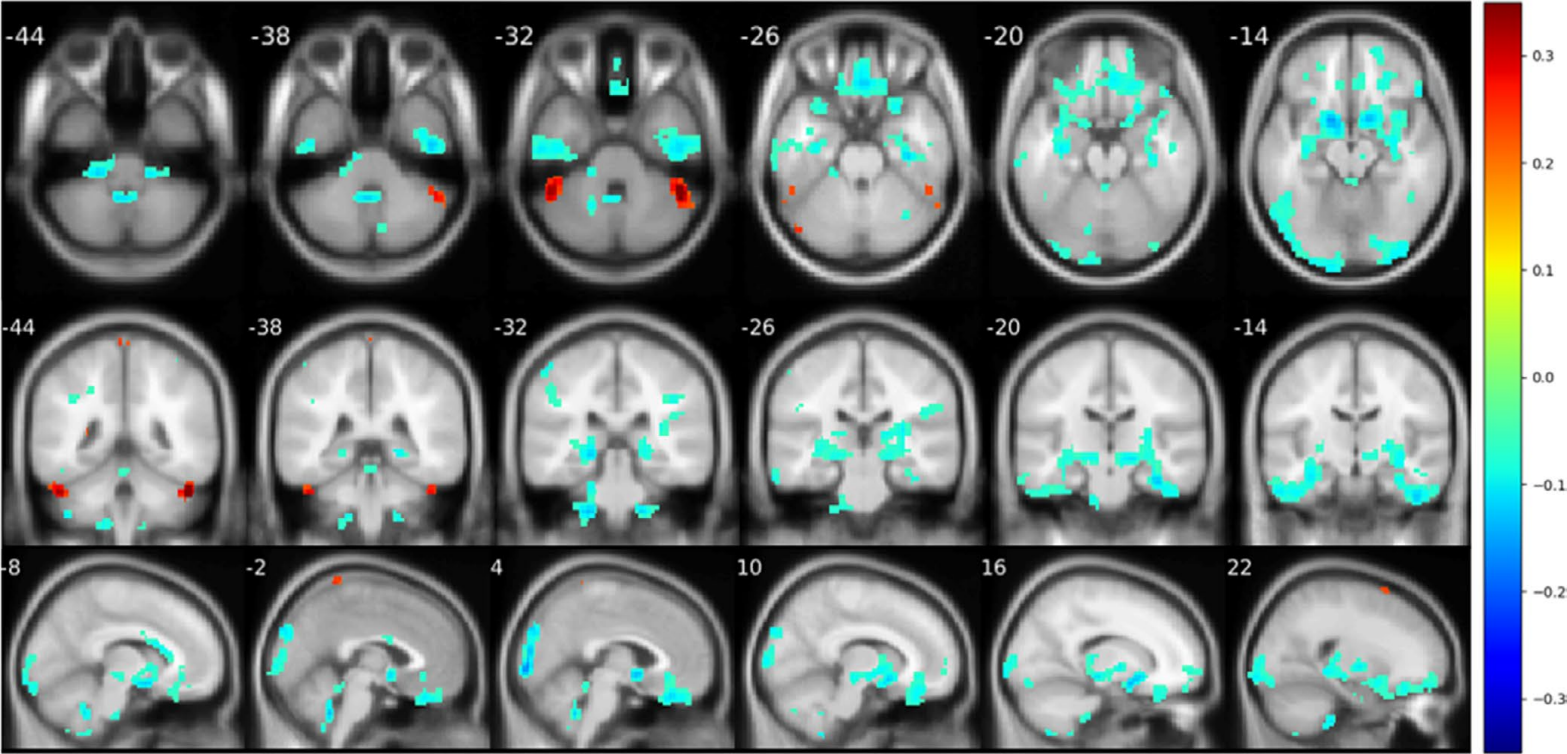
Significant difference (*p* < .05) reported from seed‐based analysis with seed in posterior lobe. Images are shown in a threshold of an absolute value 0.1 for the mean difference between two groups. Frontal cortex and occipital cortex were reported with an abnormal decline in seed‐based analysis. Furthermore, there was also a correlation decline in basal ganglia

Significant decline (*p* < .05) in cerebellum‐related connectivity was demonstrated in many brain regions of patients with OCD. For vestibulocerebellum (Figure 6), patients with OCD were associated with decreased connectivity in bilateral thalamus, posterior parts of cerebellum, and inferior occipital cortex. For spinocerebellum (Figure 7), the OCD group demonstrated a decreased connectivity in right hippocampus and rectus. For posterior lobule (Figure 8), a notable association of OCD group with a decreased basal ganglia connection including bilateral pallidum, thalamus, and putamen connections, as well as an increased connection in medial frontal cortex, was reported.

**Figure 9:**
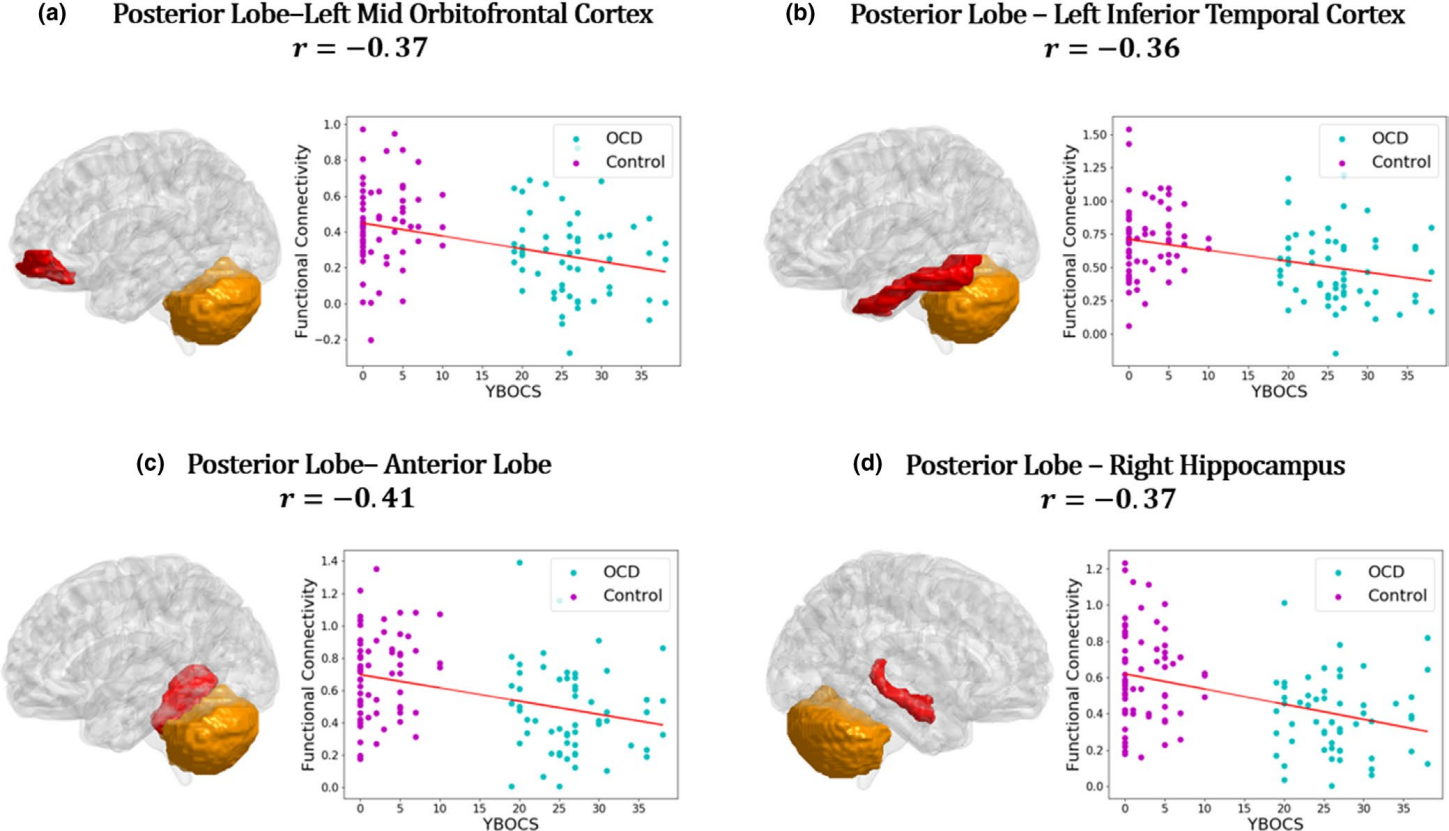
Relationship between behavioral scores and functional connectivity. Linearly fitting curves demonstrate a significant (*p* < .05) linear relationship between functional connectivity and symptom severity. Age and gender were removed as covariates. YBOCS score has a negative linear correlation with posterior cerebellar connections

Moreover, a linear regression model uses YBOCS score as predictors, covarying with age and gender, were also applied to investigate the relationships between the symptom severity and connection magnitudes. Results in Figure 9 show that symptom severity is mainly negatively correlated with the connection of the posterior parts of cerebellum.

## 4 DISCUSSION

The goal of this study was to classify patients with OCD from healthy subjects using whole‐brain functional connectivity matrices. A classifier using Riemann kernel PCA was proposed, which preserves the topological information of functional connectivity matrices and outperforms traditional classifiers such as linear PCA or other linear decomposition algorithm.

Decisive features are extracted to differentiate OCD from healthy subjects. The results revealed stronger connections between basal ganglia and cortex and weaker cerebellum‐related connections in patients with OCD. This finding is consistent with the well‐known CSTC model (Ahmari & Hen, 2013; Alexander, DeLong, & Strick, 1986; Vaghi et al., 2017). On the other hand, the cerebellum has gained significant attention in the last two decades for its influence on cognitive processes, and the functional abnormalities of cerebellum are associated with a variety of psychiatric disorders (Phillips, Hewedi, Eissa, & Moustafa, 2015; Sullivan, 2010). Some researchers have carried on explorations on its role in OCD. Kasikci, Metin, and Tas (2015) reviewed studies on cerebellar structural and functional differences in OCD and argued that cerebellum should be involved in obsessive–compulsive disorder‐related brain network model for a better understanding of the nature of this disorder. Tian et al. (2016) investigated functional connectivity strength and hubs of whole‐brain networks and found affected functional connectivity strength in the cerebellum was significantly associated with global OCD symptom severity, providing the evidence about OCD‐related brain network hub changes, not only in the CSTC circuits but more distributed in whole‐brain networks. In 2017, Bruchhage et al. (2017) investigated cerebellum and brainstem shape across two pediatric ASD and OCD cohorts in order to identify regional differences and their correlation with compulsive behavior and symptom severity and found while the anterior brainstem correlated with compulsive behaviors in both groups, larger reshaping of the cerebellum was only shown in the OCD group.

We also conducted seed‐based analyses on three functional parts of the cerebellum, a brain structure not concerned in the traditional CSTC model. The results indicated reduced connectivity in cerebellum of patients with OCD, particularly in thalamus and hippocampus, than healthy subjects. Furthermore, when using the YBOCS scores as predictors, the results showed a significant negative correlation between OCD symptom severity and the FC strength value in the posterior parts of the cerebellum. The posterior cerebellum has been found to play a significant role in neurocognition from investigations of anatomy, fMRI tasks, and clinical diseases. Stoodley and Schmahmann (2010) found that the primary motor cortex is predominantly connected to the anterior part of the cerebellum, whereas the associative cortices are predominantly connected to the posterior part of the cerebellum. Evidence from task fMRI also showed that different areas of poster cerebellum have been activated and associated with language‐related activity, working memory and reading tasks, affective processing, executive functioning, and spatial processing (Kim, Ugurbil, & Strick, 1994; Stoodley & Schmahmann, 2010; Stoodley, Valera, & Schmahmann, 2012). A clinical study by Merchant, Sharma, Xiong, Wu, and Conklin (2011) in 2014 found a significant correlation between the radiation dose to the infratentorium and posterior cerebellum and neurocognitive impairment at several cognitive domains in seventy‐eight children with low‐grade glioma. To date, this is the only available study with separate dosimetric data for the posterior cerebellum. Another study on brain injury by Schmahmann and Pandyat (Schmahmann, 1997) described the clinical cerebellar cognitive affective syndrome (CCAS) after studying 20 adults with cerebellar lesions due to either neoplasms, or vascular or traumatic damage. They all showed deficits in multiple cognitive domains including maintaining semantic and episodic memory and consciousness. Lesions of the posterior lobe of the cerebellum were produced in executive and visual‐spatial functions. In this study, the abnormal FC of the postpart cerebellum mainly focus on frontal, occipital, temporal cortex and two subcortical structures, the basal ganglia and the right hippocampus. Among them, the frontal cortex, the right OFC, and the basal ganglia have already been brought into the CSTC model. Considering that multiple cognitive damage related to OCD and the increasing mentioned voice of posterior cerebellum in cognition, more studies of posterior cerebellum need to be conducted in OCD pathomechanism.

There were some limitations to our study. First, patients with OCD were mostly treated with antidepressants and had long average illness duration, so the potential interfere of medications on the neuronal and behavioral responses cannot completely be removed in our results. Second, limited by the sample size, it is not feasible to conduct analysis based on OCD subtypes. Third, the present study is a pilot one when it comes to the finding of abnormal functional connectivity of cerebellum in OCD, considering a more accurate partition method of cerebellum. Finally, from a methodological perspective, the input data we used in the computational framework were the low‐order FC value. Further studies using different subtypes, drug‐naïve cohorts and new methods integrating time‐varying, multi‐frequency, and even multimodal information for even higher‐order functional connectivity are required.

In conclusion, this study provides a new and efficient method to characterize patients with OCD using resting‐state functional MRI. Using the property that SPD matrices could form a Riemann manifold, the proposed Riemann kernel PCA could extract features from functional connectivity matrices in an unsupervised manner. We also provide a new perspective to analyze disease‐related features. In our method, the importance of features is model‐driven, which means the importance scores are given by how useful these features are in the classifiers. Despite of CSTC circuit, our model‐driven feature analysis reported cerebellum as an OCD‐related region. This paper may provide novel insight to the understanding of genetic etiology of OCD.

## CONFLICT OF INTEREST

The authors declare no competing financial interests.

## Data Availability

The data that support the findings of this study are available from the corresponding author (Dr. Ziwen Peng, pengzw@email.szu.edu.cn) upon reasonable request.
